# Comparative Serological Assays for the Study of H5 and H7 Avian Influenza Viruses

**DOI:** 10.1155/2013/286158

**Published:** 2013-09-15

**Authors:** Eleonora Molesti, Adelaide Milani, Calogero Terregino, Giovanni Cattoli, Nigel J. Temperton

**Affiliations:** ^1^Viral Pseudotype Unit, Medway School of Pharmacy, University of Kent, Central Avenue, Chatham Maritime, Kent ME4 4TB, UK; ^2^FAO-OIE and National Reference Laboratory for Newcastle Disease and Avian Influenza, Istituto Zooprofilattico delle Venezie, Viale dell'Università 10, 35020 Legnaro, Italy

## Abstract

The nature of influenza virus to randomly mutate and evolve into new types is an important challenge in the control of influenza infection. It is necessary to monitor virus evolution for a better understanding of the pandemic risk posed by certain variants as evidenced by the highly pathogenic avian influenza (HPAI) viruses. This has been clearly recognized in Egypt following the notification of the first HPAI H5N1 outbreak. The continuous circulation of the virus and the mass vaccination programme undertaken in poultry have resulted in a progressive genetic evolution and a significant antigenic drift near the major antigenic sites. In order to establish if vaccination is sufficient to provide significant intra- and interclade cross-protection, lentiviral pseudotypes derived from H5N1 HPAI viruses (A/Vietnam/1194/04, A/chicken/Egypt-1709-01/2007) and an antigenic drift variant (A/chicken/Egypt-1709-06-2008) were constructed and used in pseudotype-based neutralization assays (pp-NT). pp-NT data obtained was confirmed and correlated with HI and MN assays. A panel of pseudotypes belonging to influenza Groups 1 and 2, with a combination of reporter systems, was also employed for testing avian sera in order to support further application of pp-NT as an alternative valid assay that can improve avian vaccination efficacy testing, vaccine virus selection, and the reliability of reference sera.

## 1. Background

Egypt faced its first H5N1 outbreak in 2006 where a highly pathogenic avian influenza (HPAI) virus was detected in poultry [1]. The strategy used by the Egyptian authorities to mitigate this relied on vaccinating poultry, depopulating infected areas, and increasing awareness and biosecurity levels. Despite these efforts, by 2008, the H5N1 virus had become endemic, and vaccine-escape variants have emerged despite commercial poultry vaccines exhibiting protection in laboratory settings [2]. For each year, from 2009 through 2012, Egypt has had more laboratory-confirmed human cases reported to the WHO than any other country, and global concern regarding Egyptian H5N1 influenza viruses is currently high, as some isolates have been reported to possess at least two mutations, of the 4 (or 5) needed to confer ferret-to-ferret airborne transmissibility [3]. Despite the mass vaccination program undertaken in poultry, the continuous circulation of the virus has resulted in a progressive genetic evolution and a significant antigenic drift with multiple mutations near the major antigenic sites [4]. To date, the WHO has identified 12 new H5N1 clades, and the Egyptian clade 2.2.1 was further split into a new subclade 2.2.1.1 [5, 6]. The past experience in Egypt has proved that controlling avian influenza in poultry is the primary method to reduce the human risk from infection and monitoring virus evolution can be extremely important for understanding the pandemic risk posed by certain subtypes, especially those prone to antigenic drift mechanisms as evidenced by the genetic and antigenic divergence of H5N1 HPAI viruses in Egypt [7–9].

Furthermore, it has been highlighted as a priority to combine vaccination with the implementation of specific systems to detect early infection with low pathogenicity avian influenza (LPAI) viruses and to study naturally acquired or vaccine-induced immunity in avian species via appropriate diagnostic tools and serological surveillance [10, 11]. Recent studies have stressed the need of reinforcing serological tests as an auxiliary tool to evaluate the potency of commercial vaccines and monitor vaccine-driven evolution of emerging variants and consequent choice of seed viruses [2]. This has been clearly recognized when the inactivated vaccine containing an H5 virus belonging to a different lineage to the Eurasian H5N1 (H5N2/Mexico) is being actively used in order to control the HPAI outbreak in Egypt from 2006 [12–14]. 

As shown by our earlier study, the emergence of an Egypt H5N1 drift variant (circulating one year later from the first H5N1 outbreak) exhibited significantly decreased cross-reactivity by haemagglutination inhibition (HI) and microneutralization (MN) assays against the Mexican vaccine seed strain [15]. This evidence, together with previous observations, has raised the important question of the mechanism of antigenic drift under vaccine pressure. Additionally, the key role of an active serological postvaccination surveillance for the assessment of vaccine efficacy and evaluation of cross-neutralizing capability of the vaccine concurrent with incremental virus escape from neutralizing antibodies is important [16].

There is currently a wide range of serological assays available for influenza; the choice is mainly based on the viral protein targeted, the level of specificity required (subtype specific or nonsubtype specific tests), and the laboratory facilities needed for certain strains [17]. Despite the complexity of the antibody response against influenza viruses, the standard serological tests such as HI and MN are routinely employed in avian influenza reference laboratories as promoted by the FAO/OIE Network of expertise on animal influenza (http://www.offlu.net/) and WHO [18]. More recently, due to their wide applicability and sensitivity, pseudotype-based neutralization (pp-NT) assays have been shown to be valid alternatives to these established methods for studying the serological profiles of highly pathogenic influenza viruses, vaccine-induced immunogenicity, and serological cross-reactivity of haemagglutinins (HAs) from different clades [19–21]. Moreover, recent studies [22, 23] have revealed that broadly cross-neutralizing antibodies binding to the stalk region of HA can be indirectly measured by HA pp-NT assays and to a lesser extent by MN but not by HI which only measures those antibodies that bind to the globular head and interfere with receptor binding [24–27]. This study reports on the screening of avian sera for antibodies elicited by LPAI and HPAI viruses and proposes a new perspective for the widening application and validation of pp-NT serological assays especially with the potential to streamline the screening of large sample sets collated from in-field seroepidemiology studies and vaccination programmes [17]. Towards this aim, we have firstly constructed HA pseudotypes from an HPAI Egyptian H5N1 virus and its closely related antigenic drift variant for a comparative serological framework to study cross-strain immunity induced by an LPAI H5N2 vaccine. Subsequently, in this study, the HA-pseudotype panel has been expanded in order to demonstrate their unique versatility (via the use of alternative reporter systems), reliability (by testing sera from naturally infected birds and reference sera) and propose them as powerful tools to support in-field and laboratory-based avian serology. 

## 2. Materials and Methods

### 2.1. Plasmids and Pseudotype Virus Production

Lentiviral pseudotypes with HA envelope glycoproteins derived from the HPAI viruses H5N1 (A/Vietnam/1194/04, A/chicken/Egypt/1709-1/2007 and A/chicken/Egypt/1709-6/2008) and H7 (A/chicken/Italy/13474/99) were constructed as described previously, with exogenous soluble neuraminidase (NA) (1 Unit/plate; Sigma) being added after transfection in order to induce the release of HA-pseudotypes from the surface of producer cells [28–30]. H5 and H7 pseudotypes were produced by cotransfection of HEK-293T cells with a complex comprising HA-expression plasmids (pl. 18-HA), the HIV type 1 *gag-pol* (pCMV-Δ8.91) and the firefly luciferase reporter constructs (pCSFLW) using Fugene 6 (Roche) that facilitates highly efficient DNA transport into cells [31–33]. Additionally green fluorescent protein (GFP) pseudotypes bearing H5 glycoproteins from A/Vietnam/1194/04 and A/chicken/Egypt 1709-1/2007 strains were generated by incorporation of GFP retroviral construct (pCSGW) as reporter [34–38]. Concurrently, a no-HA control was generated by co-transfection of producer cell lines with two plasmids, *gag-pol* pCMV-Δ8.91 and pCSFLW.

### 2.2. Serum Samples

Five panels of sera were evaluated in this study and were all provided by the FAO-OIE and National Reference Laboratory for Newcastle disease and Avian influenza, Istituto Zooprofilattico delle Venezie. Panel 1 consisted of 10 sera positive for antibodies to the LPAI H5N2 vaccine strain (A/chicken/Mexico/232/94/CPA) obtained from chickens vaccinated at 21 days of age and boosted after 3 weeks with a commercially available inactivated vaccine, which has been used in previous studies [12, 15]. Panel 2 consisted of 10 sera positive for H7 collected from turkeys during an Italian outbreak caused by an LPAI virus H7N3. Panel 3 consisted of 10 sera positive for H5 with stratified incremental HI titers (ranging from 1 : 4 to 1 : 2048) collected from chickens vaccinated with an inactivated adjuvanted H5N2 vaccine and were used for comparative firefly luciferase and GFP-pseudotype neutralization assays. In order to test for influenza HA group-specific virus neutralization, panel 4, consisting of 16 reference hyperimmune sera produced against 16 influenza subtypes (from Group 1 and Group 2), was also provided. These antisera (H1N1, H2N3, H3N8, H4N8, H5N1, H6N2, H7N3, H8N4, H9N2, H10N1, H11N9, H12N5, H13N6, H14N5, H15N9, and H16N2) were produced in specific pathogen-free chickens by inoculation with viruses (inactivated by beta-propriolactone if HPAI viruses) as described previously [12]. A panel of 41 negative sera (panel 5) confirmed by agar gel immunodiffusion assay (AGID) and Enzyme-linked immunosorbent assay (ELISA) was also employed.

### 2.3. HI and MN Assays

All the sera collected from vaccinated chickens were evaluated using standard protocols for HI and MN assays using A/chicken/Egypt/1709-1/2007 and A/chicken/Egypt/1709-6/2008 field strain antigens. HI assays were also carried out for the H5- and H7-positive serum panels using the test antigens: H5N2 (homologous to the Mexican LPAI vaccine) and H7N1 (A/Starling/Africa/985/79), respectively. Standard protocols were followed for both assays as described previously [15, 39]. For the 41 negative sera a titer of 2 was assigned when tested by HI.

### 2.4. Firefly Luciferase pp-NT Assay

For this assay, firefly luciferase pseudotypes bearing HAs from HPAI H5 (A/Vietnam/1194/04, A/chicken/Egypt/1709-1/2007,  A/chicken/Egypt/1709-6/2008) and the HPAI H7 strain (A/chicken/Italy/13474/99) were used. Two-fold serial dilutions of serum samples were mixed with an equal volume of pseudotype virus resulting in 5 × 10^5^ relative light units (RLUs) after 48 hr under standard conditions. After a 1-hour incubation at 37°C, 1 × 10^4^ HEK-293T cells were added to each well of a 96-well flat-bottomed culture plate, and RLUs were evaluated after 48 hr of incubation with a luminometer (Promega Glo Max 96) using the Bright-Glo substrate (Promega). To measure neutralization activity, the 50% and/or 90% inhibitory dose (IC_90_) was determined as the serum dilution resulting in a 50% and/or 90% reduction of a single round of infection (reporter gene-mediated signal) [28, 29, 40]. All the results were compared to control wells containing virus alone, with the RLUs from cell-only wells subtracted from all the readings. Additionally, 41 negative sera were also tested by using firefly luciferase pseudotypes (A/Vietnam/1194/04, A/chicken/Egypt/1709-1/2007, and A/chicken/Egypt/1709-6/2008).

### 2.5. GFP pp-NT Assay

The pp-NT assay was additionally performed using pseudotypes bearing HA from HPAI A/Vietnam/1194/04 and A/chicken/Egypt 1709-1/2007 strains using a packaging construct with GFP reporter gene, and the GFP pp-NT assay was essentially performed as described previously [29, 31]. In order to determine, for each strain, the amount of HA-pseudotyped virus required for this assay, complete medium was dispensed into each well of a clear 96-well flat-bottomed plate, and 8 rows of 2-fold serial dilutions of the initial virus stock were prepared, followed by the addition of 50 *μ*L of HEK-293T cells to each well. 3 days after infection, GFP expression was monitored using fluorescence microscopy. Normally 3 random fields of view are used to score the overall fraction of GFP-expressing cells, and the volume of HA-pseudotyped virus used for the assay was calculated by choosing the reciprocal pseudotype virus dilution that corresponds to the amount required to transduce 100 cells/well. The serum neutralization activity was estimated as the reduction of fluorescence expressed by the percentage of green cells in the presence of serum. Sera with no presence of neutralizing antibodies or negative sera were defined as 100% green cells or high GFP expression. 

### 2.6. Statistical Analysis

The estimation of pseudotype transduction titers was performed using Excel software where pseudotype titers obtained at each of a range of dilution points were expressed as RLU/mL, and the arithmetic mean was calculated by GraphPad Prism (version 5, GraphPad Software, San Diego, CA, USA). Statistical analyses were also undertaken for the analysis of pp-NT assays using GraphPad. Pp-NT titers were normalized, and IC_50_ and IC_90_ values were calculated by dose-response inhibition analysis. In order to assess correlation between pp-NT, HI, and MN, antibody titers were log_10_ transformed, and Pearson's correlation analysis was used.

## 3. Results 

The initial aim of the present study was to study, via a comparative serological approach, the profile, described in our earlier study [15] of influenza H5N1 subclade 2.2.1 A/chicken/Egypt/1709-1/07 virus and its antigenic drift variant belonging to subclade 2.2.1.1 A/chicken/Egypt/1709-6/08 in order to confirm the reliability of pp-NT results when employed in parallel with standard HI and MN tests. Subsequently, we investigated whether pseudotypes bearing HPAI H5 and H7 are accurately able to accurately detect neutralizing antibody responses elicited by LPAI H5 and H7 avian influenza viruses with the flexibility of using different reporter genes expressed by lentiviral vectors pseudotyped with influenza HA glycoproteins. In order to show the validity and robustness of the pp-NT method for its application to large-scale serological analyses, the results obtained by pp-NT assays were compared with HI and MN tests. 

### 3.1. Panel H5 Positive (Collected from LPAI H5N2 A/chicken/Mexico/232/94 Vaccine Trial)

Neutralizing antibodies were measured using firefly luciferase HPAI H5 influenza pseudotypes bearing HA glycoproteins derived from the HPAI viruses, clade 1 A/Vietnam/1194/04, A/chicken/Egypt 1709-1/2007, and A/chicken/Egypt/1709-6/2008. These pseudotypes were used in a neutralization assay for the detection of antibodies in a panel of 10 sera collected from chickens vaccinated with an LPAI strain belonging to a different lineage: A/chicken/Mexico/232/94/CPA (H5N2). Mexican-derived A/H5N2 inactivated vaccines were commonly used for vaccination programs in poultry farms, as undertaken in Egypt, where the samples used for this study were collected [12, 41]. A broad range of IC_90_-neutralizing antibody titers was observed in these sera, tested in duplicate against A/Vietnam/1194/04, A/chicken/Egypt 1709-1/2007, and the drift variant A/chicken/Egypt/1709-6/2008 (Table 1). 41 negative sera previously tested AI antibody free by ELISA and AGID assays (data not shown) were also found negative by H5N1 A/Vietnam/1194/04, A/chicken/Egypt 1709-1/2007, and A/chicken/Egypt/1709-6/2008 HA pp-NT. 

**Figure 1:**
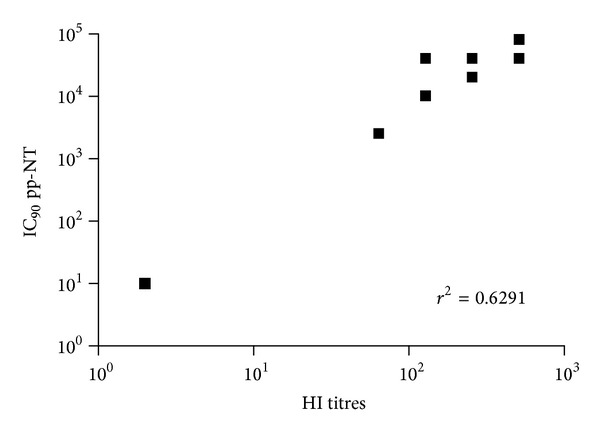
Comparison of pp-NT with HI antibody titer. Scatterplots showing the correlation of antibody logarithmic titers measured by pp-NT (using A/chicken/Egypt/1709-1/2007) versus HI (tested against A/chicken/Egypt/1709-1/2007). Correlation gave a *P* value < 0.0001.

**Figure 2:**
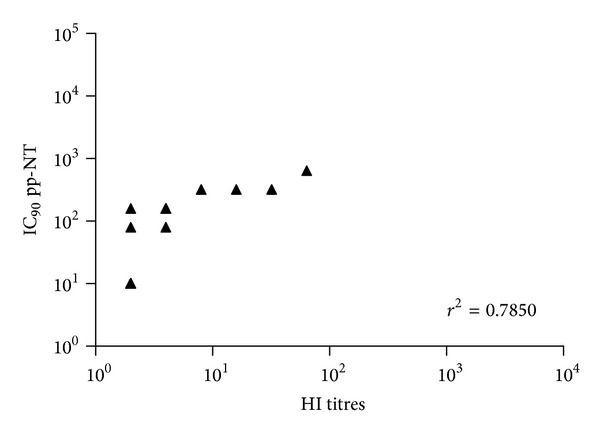
Comparison of pp-NT with HI antibody titers. Scatterplots showing the correlation of antibody logarithmic titers measured by pp-NT (using A/chicken/Egypt/1709-6/2008) versus HI (tested against A/chicken/Egypt/1709-6/2008). Correlation *P* value < 0.0001.

**Figure 3:**
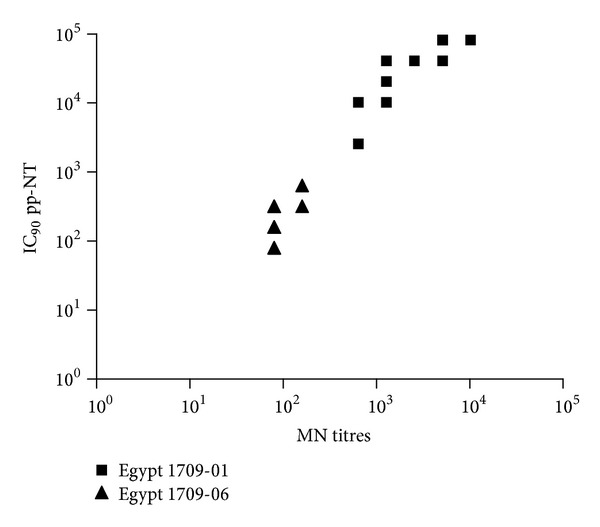
Comparison of pp-NT with MN antibody titers. Scatterplots showing the correlation of antibody logarithmic titers measured by pp-NT (using A/chicken/Egypt/1709-1/2007 and A/chicken/Egypt/1709-6/2008) versus MN (tested against A/chicken/Egypt/1709-1/2007 and A/chicken/Egypt/1709-6/2008).

In order to assess whether the results obtained with pp-NT assay mirrored those obtained with conventional assays (HI and MN) extensively used for influenza serology, a regression analysis on paired datasets was performed in order to measure the significance of correlation. The results of this analysis were supported by a highly statistically significant correlation (*P* < 0.001) between antibody titers obtained by HI, MN, or pp-NT. As shown in the scatterplots, titers obtained via HI correlated strongly with titers obtained using clade 2.2.1 A/chicken/Egypt 1709-1/2007 (*r* ^2^ = 0.6291) (Figure 1) and the drift variant A/chicken/Egypt/1709-6/2008 (*r* ^2^ = 0.7972) (Figure 2). Similar levels of correlation were observed between pp-NT titres and MN titres for both Egyptian strains (Figure 3).

**Figure 4:**
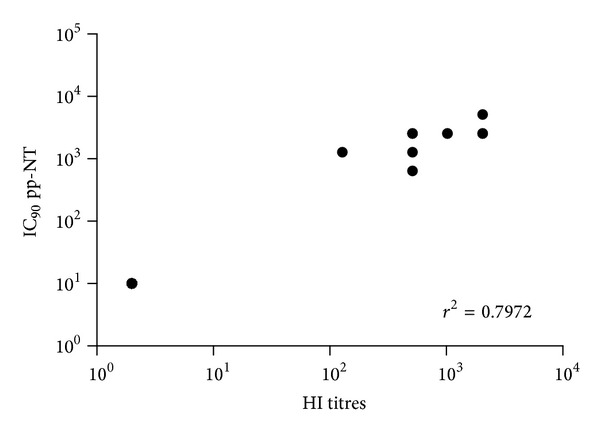
Comparison of pp-NT with HI antibody titers. Scatterplots showing the correlation of IC_90_ pp measured by pp-NT (using A/Vietnam/1194/04) versus HI (tested against A/chicken/Hidalgo/28159-232/1994). Correlation gave a *P* value < 0.0001.

Similar correlation parameters were observed between HI titers and clade 1 A/Vietnam/1194/04 pseudotype (Figure 4). 

#### 3.1.1. Incremental HI Positive H5 Serum Panel

*Measurement of Neutralizing Antibodies Using GFP and Firefly Luciferase HPAI H5 Pseudotypes.* In order to determine the reliability and the applicability of the pp-NT assay using different reporter systems, H5 A/Vietnam/1194/04 and A/chicken/Egypt 1709-1/2007 pseudotypes carrying the GFP were tested against a panel of sera positive by HI with incremental titers ranging from 1 : 8 to 1 : 2048. Three sera (3929-1, 3929-9, and 3929-6) were scored as 100% neutralization activity with pp titers >1 : 1280 (no GFP expression was observed) and sera 3930-19, 3931-26, and 3930-20 showed 50% neutralization activity at 1 : 80, 1 : 160, and 1 : 320 when tested against A/Vietnam/1194/04. IC_50_ values corresponding to titers around ≤1 : 40 were obtained for sera with HI titers lower than 1 : 32. For A/chicken/Egypt 1709-1/2007 pseudotypes, a similar pattern was observed: 4 sera (3929-1, 3929-9, 3929-6, and 3930-19) have shown complete neutralization; for 3 sera (3931-26, 3930-20, and 3933-41) 50% neutralization activity was scored between 1 : 640 and 1 : 1280. For 2 sera (3933-42 and 3933-50) percentage values of 50% lay between 1 : 80 and 1 : 320. 

In order to support the quantitative results obtained using GFP-pseudotypes, the panel of sera was tested in parallel against firefly luciferase HA pseudotype. pp-NT results were found to correlate strongly with HI showing a similar neutralization profile; however, for sera with an HI titre lower than 1 : 32, it has not been possible to determine the respective pp-NT neutralization values when H5 A/Vietnam/1194/04 pseudotypes have been used (Table 2).

### 3.2. Panel H7 Positive (Collected from an LPAI H7 Outbreak in Italy)

**Figure 5:**
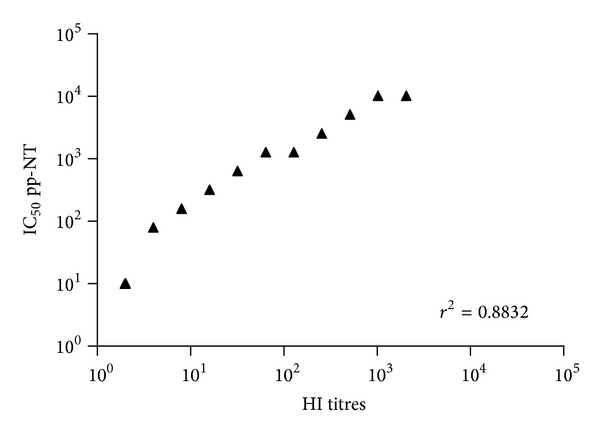
Comparison of pp-NT with HI antibody titers. Scatterplots showing the correlation of antibody logarithmic titers measured by pp-NT (using A/chicken/Italy/13474/99) versus HI (tested against A/starling/Africa/985/79 (H7N1)). Correlation gave a *P* value < 0.0001.

A panel of 10 sera collected from turkeys during an Italian epizootic caused by an LPAI H7 virus was tested by A/chicken/Italy/13474/99 HA-pseudotype assay and by HI using H7N1 (A/Starling/Africa/985/79) as antigen. All sera were positive by HI showing a panel of different titers and 10/10 closely correlated with titers obtained by pp-NT as shown in Figure 5. 41 chicken sera confirmed AI antibody free by ELISA and AGID assays were also found negative when tested by H7 A/chicken/Italy/13474/99 pseudotype-based assay.

### 3.3. Cross-Reactivity of Influenza HA Groups 1 and 2 Pseudotypes Using a Panel of Avian Reference Sera against All 16 HA Subtypes

**Figure 6:**
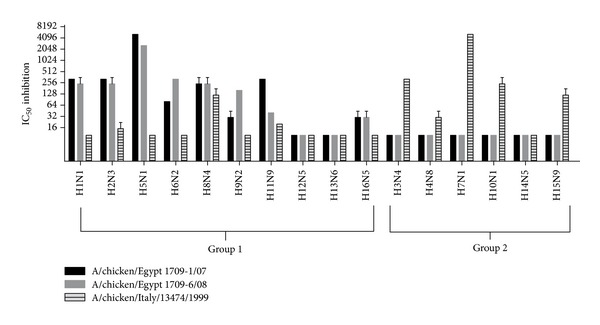
pp-NT assay showing the presence of cross-reactivity in avian reference sera between 1 and 2 influenza groups tested by H5 A/chicken/Egypt/1709-1/2008 and A/chicken/Egypt/1709-6/2008 and H7 A/chicken/13474/Italy/1999. Values corresponding to 50% neutralization (IC_50_) and with threshold serum dilution of ≥10 were considered positive.

In order to determine the extent of HA-group-specific “heterosubtypic” cross-reactivity, we tested the ability of reference hyperimmune avian sera (raised against H1N1, H2N3, H3N8, H4N8, H5N1, H6N2, H7N3, H8N4, H9N2, H10N1, H11N9, H12N5, H13N6, H14N5, H15N9, and H16N2) to neutralize pseudotypes produced with the Group 1 viruses belonging to different clades: H5 A/chicken/Egypt 1709-1/2007 and A/chicken/Egypt/1709-6/2008 and Group 2 virus: H7 A/chicken/Italy/13474/99. The quantity of H5 and H7 pseudotypes was chosen in order to have a virus input around 1 × 10^5^ RLUs, and the cross-neutralization activity for both subtypes was determined as the serum dilution resulting in 50% reduction of luciferase signal. Sera with IC_50_ titers equal to or below 1 × 10^1^ were considered not cross-reactive as shown in Figure 6. It is notable that HA-influenza pseudotypes are able to detect cross-specific neutralization within Groups 1 and 2, and, with some variation observable, H5 pseudotypes show similar patterns of cross-reactivity. Both Group 1 H5 pseudotypes (A/chicken/Egypt/1709-1/2008 and A/chicken/Egypt/1709-6/2008) exhibited cross-reactivity with sera generated against the subtypes H1N1, H2N3, H6N2, and H8N4. The Group 2 H7 pseudotype (A/chicken/Italy/13474/99) exhibited cross-reactivity with sera generated against H3N8, H4N8, H10N1, and H15N9. As additional control, H5 and H7 pseudotypes were also tested against reference sera H5N1 and H7N3, respectively, in order to provide evidence of cross-reactivity between Group 1 and Group 2 (Figure 6).

## 4. Discussion and Conclusions

Up to 1995, there had been only three reports of avian influenza viruses infecting humans, in 1959, 1977, and 1981. However, since 1996 there have been regular reports of natural infections of humans with avian influenza viruses [42]. Although these infections seem to have been limiting, with very little human to human transmission, the potential emergence of a virus capable of spread in the human population could occur via different mechanisms such as avian and human virus reassortment, recirculation of existing subtypes, and/or gradual adaptation of animal viruses to human transmission. The emergence of influenza viruses highlighted the ability of H5 and H7 subtypes to mutate from low to the highly pathogenic variant after introduction into domestic poultry [43–45]. It follows that all HPAI viruses should have an LPAI progenitor, and these incidents have raised concern about potential pandemics caused by viruses of the H5 and H7 subtypes or by any other avian influenza viruses with the potential to be transmitted to a variety of nonavian hosts including humans [44, 46–48]. In some cases mutation seems to have taken place rapidly after introduction from the wild bird reservoir; in others the LPAI virus has circulated in poultry for months prior to mutating. The factors responsible and the mechanism by which LPAI virus mutates into HPAI virus remain unclear. However, it is reasonable to assume that the wider the circulation of LPAI in poultry, the higher the chance that mutation to HPAI will occur [42]. The recent implementation of active surveillance and vaccination policies with administration of appropriate vaccines in domesticated poultry has facilitated eradication of HPAI in many countries [49]. The control of infection in poultry and the validation of more sensitive and specific assays for detecting antibodies to avian influenza viruses in avian and non-avian species represent some of the main objectives for influenza experts from the animal and public health sectors [50]. The measurement of neutralizing antibody responses is critical for influenza serodiagnosis, for the evaluation of novel vaccines and their effectiveness against drift variants arising as a consequence of vaccine pressure. The pp-NT assay represents a reliable and safe test to determine neutralizing antibody responses to all subtypes of influenza viruses [28, 51]. This neutralization assay has shown high sensitivity and specificity when compared with the established serological tests, HI and MN, and has demonstrated wide applicability for antiviral and therapeutic antibody screening and for the evaluation of vaccine efficacy. Moreover, all these methods together can be used to evaluate how well the circulating isolates match the AI vaccine formulations in order to update the vaccine by using criteria similar to those used for human influenza vaccines [52]. Exploiting the inherent sensitivity of this assay, the aim of this study was to determine the levels of antibody response against H5 and H7 pseudotypes carrying the polybasic cleavage site, in sera obtained from avian species vaccinated with commercially available inactivated vaccine that has been used in poultry farms or naturally infected with LPAI influenza viruses, and to show the correlation between pp-NT and the classical serological assays: HI and MN. 

A panel of H5-positive sera obtained from chickens vaccinated with an H5N2 A/chicken/Mexico/232/94/CPA strain was tested previously by us against the Egyptian H5N1 challenge strains (A/chicken/Egypt/1709-1/2007, A/chicken/Egypt/1709-6/2008), and significant differences between these strains have been shown by HI and MN assays, most likely due to antigenic drift driven by the implementation of vaccination in poultry [15]. In parallel, pseudotypes bearing HPAI HAs were constructed (A/Vietnam/1194/04, A/chicken/Egypt 1709-1/2007 and A/chicken/Egypt/1709-6/2008) [15, 29]. Titers obtained via HI and MN correlated strongly (Figures 1–5) with those obtained using H5 pseudotypes (for A/chicken/Egypt/1709-1/2007: *r* ^2^ = 0.62 and for A/chicken/Egypt/1709-6/2008: *r* ^2^ = 0.78). When A/Vietnam/1194/04 pseudotypes were used the correlation was *r* ^2^ = 0.79 despite the fact that the HA used in this pp-NT assay was not antigenically matched as it belongs to a different clade. The rank of ordered neutralizing values obtained by pseudotypes mirrored the HI and MN assays. Interestingly, compared with HI and MN, the pp-NT overall gives higher numerical titers and appears to be more sensitive than MN. Recent studies have raised the possibility that the lower incorporation of HA spikes into retroviral pseudotypes, compared to the wild-type virus, makes pseudotypes more sensitive allowing the binding of antibodies not only on antigenic sites of HA surface but also on the HA stalk as shown in previous studies [24, 53].

Similar results were obtained when a control panel of sera positive by HI against H7 was tested against A/chicken/Italy/13474/99 HA pseudotypes showing not only the presence of a neutralizing antibody response against HPAI H7 in sera from chickens infected by an LPAI virus but also a profile of neutralization that strongly correlates with HI. The pp-NT assay has the potential to be used in resource-limited countries where the cost-benefit of this assay could be increased by the availability of different reporter systems, for example, the use of GFP reporter instead of firefly luciferase. Additionally for laboratories lacking fluorescence or luciferase detection capability, *β*-galactosidase reporters could be used [31]. The results from this study revealed that the neutralization profile for pp-NT using a GFP reporter does not show as clearly as firefly luciferase pp-NT the titer stratification (especially for sera that give low responses by HI). A comparative analysis of results obtained using the two different reporters on the same set of sera was performed and shows a clear correlation and a strong neutralizing profile although no correlate of protection has been yet established for pp-NT assay (Table 2) [28]. 

Results for cross-reactivity analyses of Groups 1 and 2 HA influenza pseudotypes against antisera from all 16 HA subtypes shed new light on the performance of the pp-NT assay using HI standards. Firstly, the specificity that can be gained by the use of influenza pseudotypes considering that the cut-off for negative sera was assigned for IC_50_ values equivalent or below 1 × 10^1^, and H5 and H7 pseudotypes showed some degree of cross-reactivity with sera generated from viruses belonging to the same HA groups and strong reactivity when H5 and H7 pseudotypes were tested against H5-H7 hyperimmune sera as shown in Figure 6.

Furthermore, reactivity observed validates the reliability and the quality of OIE-FAO reference sera that represents a prerequisite for the improvement of sero-diagnosis and can help to evaluate the effectiveness of vaccine strategies bearing in mind that an extensive library of reference sera for all influenza strains is an essential aspect for pandemic influenza preparedness [54]. It is likely that a new panel of reference sera will need to be prepared for use with pseudotype-based assays as they become more widely used in the future.

The pp-NT assay is a valid surrogate for the more complex and time-consuming MN and for HI. Influenza pseudotypes can be employed to screen antibody responses on the particle surface due to the fact that HA is the major antigen of the virus against which neutralizing antibodies are produced [55]. It will permit HA subtyping, antigenic tracking of virus evolution, and help to improve both the evaluation of vaccine effectiveness and vaccine virus strain selection. 

## Figures and Tables

**Table 1:** IC_90_-neutralizing antibody titres tested by pseudotype-based neutralization assays for chickens immunized with the Mexican-derived H5N2 strain.

IC_90_-neutralizing antibody titres			
Sera no.	A/Vietnam/1194/04	A/ck/Egypt 1709-1/2007	A/ck/Egypt 1709-6/2008
4822/V09-1	2560–5120	>81920	160–320
4822/V09-3	2560–5120	20480–40960	80–160
4822/V09-4	320–640	1280–2560	40–80
4822/V09-5	2560–5120	10240–20480	80–160
4822/V09-6	2560–5120	20480–40960	320–640
4822/V09-7	2560–5120	>81920	640–1280
4822/V09-8	1280–2560	40960–81920	80–160
4822/V09-9	1280–2560	>81920	320–640
4822/V09-10	2560–5120	5120–10240	80–160
4822/V09-12	2560–5120	40960–81920	40–80

Range of titres observed	(320–5120)	(1280–81920)	(40–1280)

**Table 2:** Comparison between pp-NT assays using different reporter systems (GFP and CSFLW luciferase) and HI tests. IC_50_-neutralizing antibody titres tested by A/Vietnam/1194/04 and A/Egypt 1709-01/07.

Sera no.	HI	IC_50_ A/Vietnam/1194/04 GFP-pp	IC_50_ A/Vietnam/1194/04 Luc-pp	IC_50_ A/Egypt 1709-01/07 GFP-pp	IC_50_ A/Egypt 1709-01/07 Luc-pp
3933-42	1 : 8	<40	80–160	80–160	80–160
3933-50	1 : 16	<40	640–1280	160–320	80–160
3933-41	1 : 32	<40	640–1280	320–640	1280–2560
3930-20	1 : 64	160–320	2560–5120	640–1280	2560–5120
3931-26	1 : 128	80–160	640–1280	1280	5120–10240
3930-19	1 : 256	40–80	2560–5120	>1280	5120–10240
3929-6	1 : 512	>1280	>10240	>1280	2560–5120
3929-9	1 : 1024	>1280	5120–10240	>1280	>10240
3929-1	1 : 2048	>1280	>10240	>1280	>10240
